# Restoring Balance to the Outer Membrane: YejM’s Role in LPS Regulation

**DOI:** 10.1128/mBio.02624-20

**Published:** 2020-12-15

**Authors:** Brent W. Simpson, Martin V. Douglass, M. Stephen Trent

**Affiliations:** a Department of Infectious Diseases, The University of Georgia, College of Veterinary Medicine, Athens, Georgia, USA; b Department of Microbiology, The University of Georgia, College of Veterinary Medicine, Athens, Georgia, USA; National Cancer Institute

**Keywords:** LapA, LapB, LpxC, PbgA, YejM, cardiolipin, cell envelope, lipopolysaccharide, outer membrane

## Abstract

Gram-negative bacteria produce an asymmetric outer membrane (OM) that is particularly impermeant to many antibiotics and characterized by lipopolysaccharide (LPS) exclusively at the cell surface. LPS biogenesis remains an ideal target for therapeutic intervention, as disruption could kill bacteria or increase sensitivity to existing antibiotics. While it has been known that LPS synthesis is regulated by proteolytic control of LpxC, the enzyme that catalyzes the first committed step of LPS synthesis, it remains unknown which signals direct this regulation. New details have been revealed during study of a cryptic essential inner membrane protein, YejM. Multiple functions have been proposed over the years for YejM, including a controversial hypothesis that it transports cardiolipin from the inner membrane to the OM. Strong evidence now indicates that YejM senses LPS in the periplasm and directs proteolytic regulation. Here, we discuss the standing literature of YejM and highlight exciting new insights into cell envelope maintenance.

## Abstract (precis)

Gram-negative bacteria produce an asymmetric outer membrane (OM) that is particularly impermeant to many antibiotics and characterized by lipopolysaccharide (LPS) exclusively at the cell surface. LPS biogenesis remains an ideal target for therapeutic intervention, as disruption could kill bacteria or increase sensitivity to existing antibiotics.

## PERSPECTIVE

The cell envelope of Gram-negative bacteria provides a robust barrier against antibiotics, bacteriophages, and immune cells (1). Included in the cell envelope is a symmetrical inner membrane (IM) composed of glycerophospholipids (GPLs) and an asymmetrical outer membrane (OM) composed of GPLs in the inner leaflet and primarily lipopolysaccharide (LPS) in the outer leaflet (2). LPS consists of three groups attached covalently to one another: the lipid A glycolipid, the core oligosaccharide, and a variable O-antigen that is not essential for viability (3). The cell coordinates the synthesis of both GPLs and LPS to maintain cell envelope integrity, as improper balance between GPLs and LPS results in a compromised membrane barrier (4).

**FIG 1:**
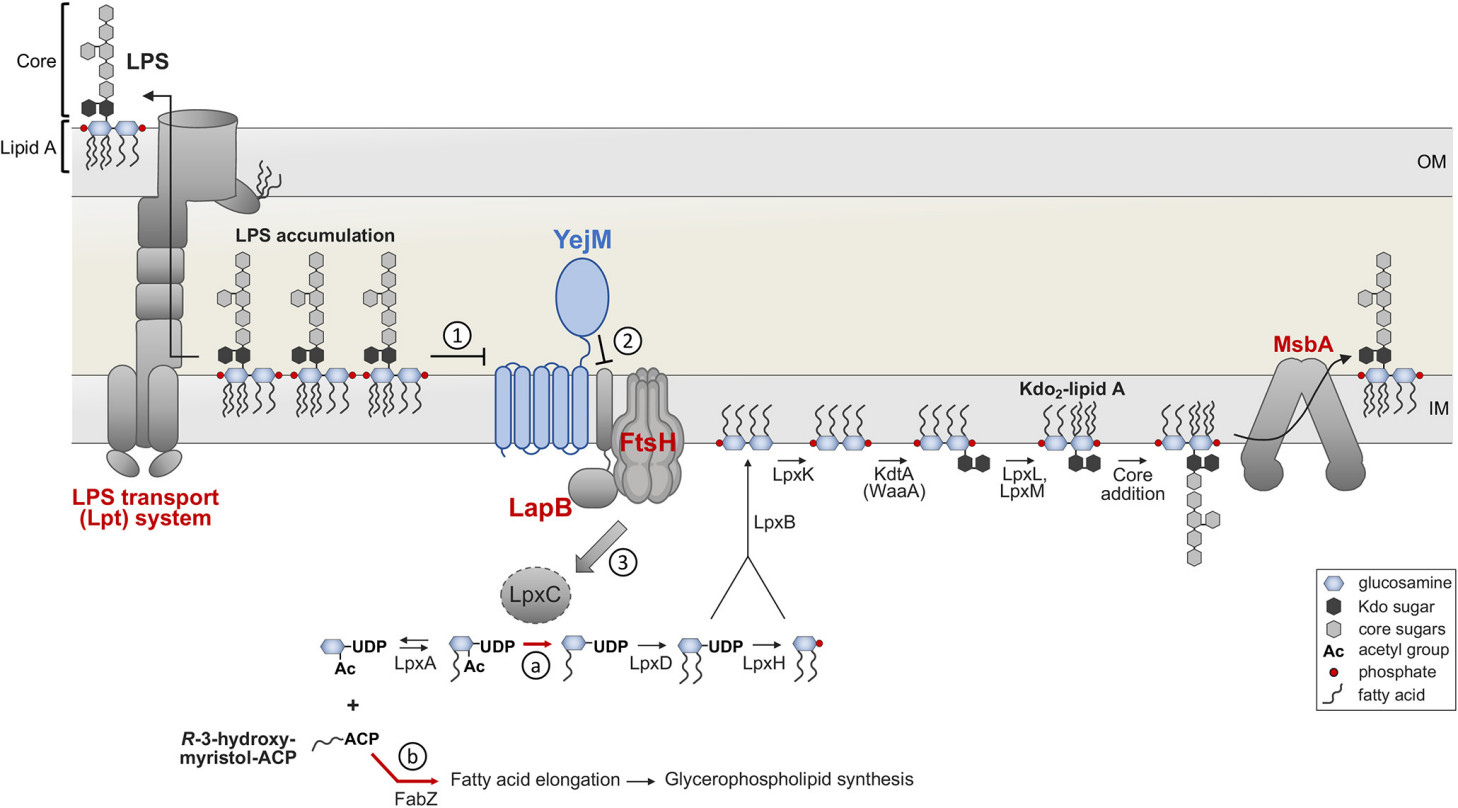
Modulation of LPS biosynthesis by YejM. The common precursor *R*-3-hydroxymyristol-ACP is used for both LPS synthesis and GPL synthesis in the cytoplasm. LPS synthesis begins with the synthesis of the Kdo_2_-lipid A subdomain that serves as the lipid anchor of LPS in the outer membrane. In E. coli, nine enzymes are required for Kdo_2_-lipid A synthesis, which begins with the acylation of UDP *N*-acetylglucosamine (UDP-GlcNAc) by LpxA. The second step of the pathway is catalyzed by LpxC, a deacetylase, and is the first committed step for lipid A synthesis. Thus, flux of fatty acids into each lipid synthesis pathway is tightly balanced through regulation of (a) LpxC which commits fatty acids to LPS synthesis and competes with (b) FabZ which continues fatty acid elongation, committing fatty acids to GPL synthesis. The canonical glucosamine disaccharide of lipid A is formed by LpxB, an inverting glycosyl transferase that condenses two di-acylated precursors to form the first disaccharide intermediate. The final steps include the addition of a second phosphate group by LpxK, incorporation of the characteristic Kdo sugars by KdtA (WaaA), and additional acylation events catalyzed by LpxL and LpxM. The core oligosaccharide is extended in the cytoplasm and then the dedicated LPS flippase, MsbA, transports core-lipid A to the periplasmic leaflet of the inner membrane (IM). When present, the long-chain O-antigen polysaccharide is added in the periplasm but is not shown here for simplicity. Translocation of complete LPS to the outer membrane (OM) is carried out by the LPS transport (Lpt) system that bridges the IM and OM. The membrane protease FtsH, along with the adaptor protein LapB, degrades LpxC and thus controls the rate of LPS synthesis and provides metabolic adaptation to various growth conditions. When the need for LPS is high, YejM is bound to LapB, thereby preventing proteolytic degradation of LpxC by FtsH. However, if LPS begins to accumulate within the periplasm (1), YejM binds to LPS to disrupt YejM-LapB interactions (2). This allows for FtsH/LapB degradation (3) of LpxC, thus disrupting lipid A synthesis and lowering LPS levels. As noted by Clairfeuille and coworkers (14), in this model YejM functions as a “rheostat” to regulate LpxC levels.

The synthesis of the OM is synchronized by an *R*-3-hydroxymyristol acyl carrier protein (ACP) that serves as an acyl donor for both GPL and LPS synthesis (Fig. 1). FabZ, a dehydratase, utilizes *R*-3-hydroxymyristol-ACP in fatty chain elongation (5). Elongated fatty acid chains are then utilized for GPL synthesis, whereas LpxA utilizes *R*-3-hydroxymyristol-ACP in the first step in lipid A synthesis (Fig. 1) (6). Because LpxA catalyzes a reversible reaction, the control point of substrate flux into GPL and LPS synthesis is at LpxC, the second and irreversible step of lipid A synthesis (6). The cell utilizes FtsH, an ATP-dependent zinc metalloprotease located at the IM, to degrade LpxC to control flux of lipid precursors into lipid A biosynthesis (4). In tandem with FtsH, the accessory protein LapB (YciM) and possibly LapA (YciS) aid in the degradation of LpxC (7–9) (Fig. 1, LapA is not shown). Which signals activate the LapAB/FtsH regulation and how this regulation mechanism works are still being elucidated. Recent advances in cell envelope biology have shed light on a third protein involved in LpxC regulation, YejM (PbgA), which directly senses periplasmic LPS levels (10–14). Discovery of this third member in this regulatory cascade to control LpxC levels will help to finally unravel how bacteria balance GPL and lipid A synthesis and respond to changes in each. As lipid A synthesis is essential and critical for pathogenesis in most Gram-negative pathogens (15), LpxC regulation through YejM/LapAB/FtsH will be an excellent target for future antimicrobial development.

## MYSTERY ESSENTIAL PROTEIN YejM, PROPOSED FUNCTIONS OVER TWO DECADES

Characterization of a YejM mutant was first reported in 1997 by Hirvas and colleagues by subjecting an Escherichia coli K-12 strain to mutagenesis and screening for sensitivity to the hydrophobic drug fusidic acid (16). The screen generated mutant strain LH530 that was sensitive to an array of antibiotics, had decreased LPS levels, displayed temperature sensitivity, and formed filaments (16). In an effort to identify the mutant gene, a chromosomal gene bank was utilized to complement LH530 defects. Although the *yejM* mutation was not identified through this method, the LH530 strain was demonstrated to be rescued by the overexpression of AcpT, a holo-acyl carrier protein synthase (16).

It was not until 10 years later that the Cronan laboratory identified the strain LH530 to carry a mutation in *yejM* (17). YejM was identified as an IM protein consisting of 586 amino acids and containing 5 essential transmembrane helices and a large but dispensable periplasmic domain (17) (Fig. 1). YejM was truncated to 189 amino acids in the LH530 strain, resulting in loss of the periplasmic domain (17). While this work did not identify YejM’s essential role in the cell, it did determine that overexpression of AcpT could even rescue cells harboring a complete deletion of YejM (17).

Almost 10 years passed until YejM resurfaced in the scientific literature. In 2015, Miller and colleagues conducted a screen in Salmonella enterica serovar Typhimurium to search for mediators of PhoPQ remodeling of the OM (18). *Salmonella* rely on the PhoPQ two-component system to remodel the OM to increase the barrier function. Five unique insertions were identified in *yejM*, each truncating the periplasmic domain, which led to increased membrane permeability when PhoPQ was activated (18). It was hypothesized that YejM is critical for changes to the OM barrier controlled by PhoPQ, so it was renamed from YejM to PbgA (PhoPQ-Barrier Gene A) (18). Previously, the Miller group reported that increased PhoPQ activity caused increased accumulation of acidic GPLs in the OM, such as cardiolipin (19). When the *yejM* mutant was assessed, however, cardiolipin levels were not increased in the OM in response to PhoPQ activation. Therefore, it was hypothesized that YejM was involved in cardiolipin shuttling to the OM (18).

Three groups have reported crystal structures of YejM, a structure of the soluble periplasmic domain by Dong and colleagues (20) and two structures of the whole protein by the Miller group (21) and by Rutherford and coworkers (14). Two of the structures of YejM were interpreted in the possible context of YejM as a cardiolipin transporter (20, 21), while the most recent structure provided strong evidence that YejM senses and regulates LPS synthesis, as discussed below (14). The periplasmic domain of YejM is highly homologous to the arylsulfatase family, which includes enzymes that interact with lipids, such as lipoteichoic acid synthase (LtaS), and is more distantly related to the lipid A phosphoethanolamine transferases, which include EptA and the MCR (mobile colistin resistance) gene family (20). However, YejM apparently lacks proper placement of catalytic residues, suggesting it does not possess enzymatic activity (14, 20). Whole-protein structures of YejM by Miller and coworkers showed the protein was bound to two cardiolipins (21), while the whole-protein structure reported by Rutherford and colleagues was bound to only LPS (14). Notably, the bound cardiolipin molecules were supplemented during crystallization (21), whereas the bound LPS was copurified with YejM (14). The proposed role for YejM in cardiolipin transport has remained controversial and will be discussed later. However, recent work from five groups has provided clear evidence that YejM functions in regulation of lipid A synthesis.

## EMERGING ROLE IN LPS REGULATION

A common theme in early research on YejM was that mutations that disrupt YejM caused increased sensitivity to antibiotics and changes to the OM. Both of these points hint at *yejM* mutants having a disrupted OM permeability. The Misra group also came to this conclusion while studying iron uptake (22). Starting with E. coli mutants lacking TonB, an IM protein that energizes iron uptake (23), the group searched for suppressors able to utilize exogenous iron. Suppressors were identified that resulted in truncation of the periplasmic domain of YejM (22). *yejM* mutants were found to have a general increased permeabilization of the OM, allowing iron to enter the cell (22).

Shortly after, the Dalebroux group found the first link for YejM to LPS stability and synthesis (11). Mice were challenged with *Salmonella* mutants that produced a truncated YejM, lacking the periplasmic domain. Although the mutant strain was highly defective for virulence in mice, *yejM* suppressor mutants survived the challenge and were isolated (11). These suppressors contained point mutations in *lpxC*, *ftsH*, or *lapB*, all proteins involved in LPS synthesis and regulation, paving the way toward identifying a bona fide function for YejM (11).

Three consecutive papers from the Silhavy (10), Bernhardt (12), and Misra (13) laboratories revealed the essential role of YejM in E. coli through suppressor analysis. The Misra group began by generating suppressors to large antibiotics (erythromycin and vancomycin) in a hypersensitive E. coli mutant that produces a periplasmic-truncated YejM (13). These suppressors contained mutations in *lapB* and *ftsH*. Since both of these genes are involved in LpxC regulation, they next measured LpxC levels. The YejM truncation was demonstrated to decrease LpxC levels and thus lipid A/LPS synthesis (13). All suppressors isolated by Misra and coworkers had increased LpxC stability, restoring lipid A synthesis. Additionally, suppressors in *lapB* led to undetectable levels of LapB (13), resulting in increased levels of LpxC and indicating a strong negative correlation between the two proteins.

Furthermore, Bernhardt and colleagues isolated viable suppressors in E. coli lacking the complete YejM protein, and these suppressors were mapped to *lpxC* and *lapB* (12). Like the Misra laboratory, they also revealed that stabilizing LpxC levels rescued *yejM* mutants (12). The previously characterized suppression of *yejM* mutants by AcpT overexpression was also tested, and showed similarly stabilized LpxC levels (12). This finding indicated that AcpT suppression also acts on LpxC stability, but the mechanism requires further investigation. If AcpT is a substrate of FtsH, as Bernhardt and colleagues hypothesized, than overexpression could be sequestering FtsH, causing LpxC levels to increase. Alternatively, AcpT overexpression could affect fatty acid synthesis, which may then feed into LpxC regulation (discussed more below). Further, YejM was found to interact with and bind to LapB in a two-hybrid system optimized for membrane proteins (12). Finally, the Bernhardt group demonstrated that deletion of *lapB* will allow the absence of normally essential YejM (12).

The Silhavy group also isolated suppressors of truncated *yejM* mutants, but on SDS/EDTA-containing media or at 42°C. Suppressors were mapped to *lapB* and *lpxC* (10). LPS levels were measured instead of LpxC stability, demonstrating that YejM truncations lower LPS levels and that the LPS levels are restored in the suppressor mutants (10). Together, these parallel approaches have finally determined the essential role of YejM. They have demonstrated that YejM functions with LapB to modulate FtsH turnover of LpxC, which catalyzes the first committed step of lipid A/LPS synthesis.

## PROPOSED SIGNALS FOR YejM/LapAB/FtsH REGULATION OF LpxC

FtsH-mediated regulation of LpxC has been proposed to be responsive to many signals, including LPS precursors (24, 25), coenzyme A (CoA)-linked or ACP-linked fatty acids (4, 26), stringent response (27), heat shock (8, 9), and cell cycle regulation (27). However, many questions remain for how these signals are sensed. The identification of YejM as a missing component that contributes to LpxC regulation opens up new possibilities for how the cell senses key signals and the mechanism of the regulation cascade. Together YejM, LapB, and the possible accessory protein LapA have protein domains in the cytoplasm, IM, and the periplasm. The topology of each of these components may give clues to which protein is most likely to sense cytoplasmic, intermembrane, and periplasmic signals. YejM has an essential transmembrane domain and a nonessential large periplasmic domain, whereas LapB and LapA have large cytoplasmic domains in addition to transmembrane segments.

**Sensing LPS synthesis.** Two hypotheses have been proposed for how LPS synthesis could be sensed for LpxC regulation. The first hypothesis proposed that the completed minimal product of LPS, Kdo_2_-lipid A (Fig. 1), was the signal for LpxC regulation. The second proposed that an intermediate of the lipid A synthesis pathway is sensed to direct proteolysis of LpxC. Genetic and biochemical evidence outlined below support both hypotheses. Recent work by Rutherford and colleagues has mechanistically demonstrated that completely synthesized lipid A is sensed by YejM, further supporting the first hypothesis. How lipid A intermediates may affect LpxC regulation will require further investigation.

Raetz and colleagues were the first to demonstrate that LpxC protein levels are regulated in response to the activity of other enzymes involved in lipid A synthesis (24, 28, 29). For example, the group found that point mutations that reduce the activity of the essential acyltransferases LpxA (24, 28) or LpxD (29), which act early in lipid A synthesis (Fig. 1), result in increased LpxC protein levels. Similarly, reduction of LpxC activity by addition of targeted inhibitors (24) resulted in increased protein levels and thus the specific activity of LpxC in cell extracts. The Raetz group proposed that perhaps the final synthesis product, Kdo_2_-lipid A, was acting as a signal for translational or protease regulation of LpxC (24).

However, lipid A intermediates may instead be the signal sensed by the LPS regulation machinery. For example, inhibition of synthesis of the Kdo (3-deoxy-d-*manno*-oct-2-ulosonic acid) sugars does not result in increased LpxC protein levels (24), even though Kdo addition is required for final acyl chain addition and completion of lipid A synthesis (30) (see Fig. 1). Along with previous biochemical data detailing how LpxC is upregulated in response to inhibition of key Lpx enzymes, quantitative modeling was used to predict changes in precursor pools in response to stepwise inhibition throughout the pathway, supporting the idea that the LpxB reaction product, the first intermediate showing the characteristic lipid A disaccharide backbone, may be sensed by the cell (25). This was supported by the fact that overexpression of LpxK, the enzyme utilizing the LpxB reaction product, leads to increased levels of the LpxC protein (25).

Recently, Rutherford and colleagues have provided strong evidence that YejM is a sensor for lipid A (14). The group reported a crystal structure of YejM that had additional density that fit a bound lipid A molecule. The lipid A-binding domain of YejM constituted a unique motif with 14-point interactions to an acyl-chain, an *N*-acetylglucosamine of the lipid backbone, and a phosphate group. Structure-function analysis of mutants that disrupt the lipid A-binding domain, found that introduction of negative charges into the protein structure that should repel lipid A were the most detrimental and resulted in disruption of the OM barrier. Peptides were designed to mimic the YejM lipid A-binding domain and were found to interact specifically with lipid A, but not with GPLs, including cardiolipin. Furthermore, these lipid A-binding peptides derived from YejM were toxic to E. coli and other Gram-negative bacteria, indicating they bind LPS *in vivo* and may be developed into effective therapeutics. Copurifications of YejM identified two IM interacting partners, including PlsY (involved in GPL synthesis) and LapB, supporting YejM’s role in regulating LpxC in concert with LapB. Finally, periplasmic sensing of LPS by YejM was demonstrated *in vivo*; trapping LPS on the cytoplasmic side of the IM by treating with MsbA inhibitors resulted in increased LpxC levels, whereas trapping LPS on the periplasmic leaflet by treating with Lpt inhibitors decreased LpxC levels. Thus, data from Rutherford and colleagues provide a clear view that periplasmic LPS is sensed by YejM to direct LapB regulation of LpxC (Fig. 1). Sensing LPS at the periplasmic side of the IM provides both a means to monitor LPS synthesis and transport across the IM by MsbA. Whether additional intermediates of lipid A are also sensed on the cytoplasmic side of the IM requires further attention.

**Sensing fatty acids and OM permeability.** The balance between GPL and LPS synthesis is influenced by competing activities of FabZ, which commits fatty acids to GPL synthesis, and LpxC, which commits fatty acids to LPS synthesis (Fig. 1). This was demonstrated by several groups when loss of function *fabZ* suppressors restored the balance of GPL synthesis in strains with decreased LPS levels resulting from temperature-sensitive mutation in *lpxA* or a partial-loss-of-function mutation in *lpxC* (5, 31). In addition, gain-of-function *fabZ* suppressors were found to restore the balance of GPL synthesis in a strain with increased LPS levels resulting from an *ftsH* mutation (4). Further, FtsH degradation of LpxC has been shown to respond to differences in growth rate in a FabZ-dependent manner (27). LpxC was found to be stabilized (have a longer half-life) during rapid growth and destabilized (have a shorter half-life) during slow growth (27). Overexpression of FabZ abolished LpxC regulation in response to growth rate (27), suggesting that the acyl-ACP pools were being sensed. Whether acyl-ACP pools are sensed directly to exert this balance through modulation of LpxC levels has yet to be determined. However, evidence that LpxC might be regulated in response to acyl-CoA pools was found while studying maintenance of OM asymmetry (26).

The asymmetry of the OM, i.e., the presence of LPS on the outer leaflet and of GPLs on the inner leaflet, is key to its barrier function (32). As such, Gram-negative bacteria have developed at least three methods to ensure that GPLs are not mislocalized to the outer leaflet of the OM. The Mla (maintenance of OM lipid asymmetry) transporter has been shown to transport mislocalized OM GPLs back to the IM (33), and the OM enzymes PldA (phospholipase A) and PagP (acyl transferase, transferring acyl chain from GPLs to LPS) degrade OM-mislocalized GPLs (34, 35). May and Silhavy found that when GPLs were mislocalized to the outer leaflet of the OM, LpxC protein levels and LPS synthesis were upregulated in a PldA-dependent manner (26). These findings suggested that PldA activity was producing a signal that in turn affected LpxC regulation. PldA degrades outer leaflet GPLs and lyso-GPLs to produce glycerophosphodiesters and free fatty acids that can be imported and recycled into lipid synthesis. The group found that disrupting fatty acid uptake through FadD, an acyl coenzyme A (acyl-CoA) synthetase for fatty acid recycling, prevented the upregulation of LPS synthesis (26). Together, these results support the idea that cytoplasmic acyl-CoA pools are sensed to regulate LpxC levels. In agreement, deletion of *pldA* in a YejM-truncated mutant results in a synthetic sick phenotype, supporting that both proteins impact LpxC regulation (22). This regulation could be a means of both fine-tuning GPL and LPS synthesis and sensing when the OM is perturbed through PldA-derived fatty acids in order to restore OM asymmetry. Which protein senses acyl-ACP or acyl-CoA pools has yet to be determined but is likely to occur through a cytoplasmic domain, which makes LapB or LapA possible candidates.

**Upstream regulation of YejM/LapAB.** While each of the proteins are produced basally, LapAB have been demonstrated to be upregulated by heat shock in E. coli (8, 9). Upstream regulation points may be key to fine-tuning LpxC levels, as *lapAB* mutants are sensitive to heat shock (8, 9). Finally, FtsH proteolysis of LpxC has been found to respond to stringent response (27). How levels of the alarmone ppGpp affect either the levels of the YejM/LapAB/FtsH regulators or LpxC directly has yet to be determined.

## REASSESSING THE ROLE OF YejM IN CARDIOLIPIN BIOGENESIS

With the discovery of YejM’s function in LpxC regulation, it is unlikely that YejM is also a cardiolipin transporter. While it is still possible that YejM has two functions, regulating LpxC and transporting cardiolipin, there are several lines of evidence that argue against this interpretation. YejM has always been an odd candidate for a cardiolipin transporter because it lacks an obvious OM partner. All other complexes (SecYEG/BamABCDE, LolABCDE, and LptABCDEFG) known to transport substrates from the IM to the OM (OM beta barrels, lipoproteins, and LPS, respectively) have IM, periplasmic, and outer membrane components (36–38). Still, it could be possible that additional components have yet to be identified.

One could imagine that a cardiolipin transporter might also regulate LpxC levels in order to make sure that OM GPLs and LPS were balanced. However, YejM regulation of LpxC is essential (12) while cardiolipin synthesis is not (39). If YejM were assessing cardiolipin transport, then lack of cardiolipin would be expected to highly activate YejM to toxic levels. This is not the case for mutants lacking all three of the cardiolipin synthases (40). Further, Misra and colleagues determined that YejM has a major impact on OM permeability and that *yejM* mutants allowed iron to enter the cell independently of the iron transporter (22). However, the group showed that cardiolipin-deficient strains do not recreate these phenotypes (22). Together, these lines of evidence show that cardiolipin synthesis does not affect LpxC regulation by YejM, making it unlikely that YejM is sensing cardiolipin synthesis or transport.

Finally, one could imagine a scenario where the IM domain of YejM was involved in LpxC regulation, while the periplasmic domain had an independent role in cardiolipin transport. However, truncations of the YejM periplasmic domain confer OM permeability defects and these defects can be suppressed by changes to LpxC stability (10, 13). This indicates that both the IM and periplasmic domain of YejM are involved in the same role of regulating LpxC proteolysis.

The effect that the Miller group saw on OM cardiolipin levels is still an intriguing phenomenon that requires alternative interpretations. It is possible that YejM is involved in the regulation of other targets in addition to LpxC. It is currently not clear if YejM/LapAB are dedicated only to effecting LpxC proteolysis by FtsH or if they may direct FtsH to other targets as well. Notably, the Kdo transferase involved in lipid A synthesis, KdtA (WaaA), has also been shown to be regulated by FtsH proteolysis (41). The signals and mechanism of this regulation are unclear. Alternatively, the effect on cardiolipin levels could be a downstream effect of the altered lipid A synthesis. Alterations of lipid A synthesis could be sensed by a different regulator which could then regulate either cardiolipin synthases or a true cardiolipin transporter. A final alternative hypothesis is that *yejM* mutants may have increased OM vesiculation due to the alteration in LPS levels. It has been demonstrated that disrupting the OM asymmetry, such as with mutations in genes encoding the Mla system, increases OM vesiculation (42). Since *yejM* mutants also have disruption of the OM barrier, it is possible that OM vesiculation is increased, resulting in loss of OM GPLs. The effect would be most notable on cardiolipin, as it is produced at a lower concentration then the other GPLs. Altogether, further assessment of how YejM affects OM cardiolipin may give new clues to how GPL and LPS levels are balanced.

## FUTURE DIRECTIONS

Recent lines of evidence from multiple groups have finally determined the function of the essential IM protein YejM. It is now clear that YejM directly senses periplasmic LPS to signal through LapB and regulate LpxC proteolysis. Many questions remain for how this essential regulator functions. What others signals are sensed and through which protein? How do these proteins direct FtsH proteolysis? Does YejM/LapAB affect regulation of other FtsH substrates? How do YejM mutants affect OM cardiolipin levels? How conserved is YejM monitoring of LPS synthesis? Since YejM has homology to phosphoethanolamine-lipid A transferases (like EptA) and bacterial arylsulfatases, YejM homologs would have to be carefully assessed to determine their function. Further analysis on these families of related proteins may reveal signatures that define their true function in the bacterial cell. In addition, future work on YejM will address major outstanding questions in cell envelope biology for how GPL and LPS synthesis are regulated and balanced.
